# Genome-wide association study of infectious bovine keratoconjunctivitis in Angus cattle

**DOI:** 10.1186/1471-2156-14-23

**Published:** 2013-03-26

**Authors:** Kadir Kizilkaya, Richard G Tait, Dorian J Garrick, Rohan L Fernando, James M Reecy

**Affiliations:** 1 Department of Animal Science, Iowa State University, Ames, IA 50011 USA; 2 Department of Animal Science, Adnan Menderes University, Aydin, 09100 Turkey; 3 Institute of Veterinary, Animal and Biomedical Sciences, Massey University, Palmerston North, New Zealand

**Keywords:** Keratoconjunctivitis, Pinkeye, BayesB, Threshold model, Genome-wide analysis

## Abstract

**Background:**

Infectious Bovine Keratoconjunctivitis (IBK) in beef cattle, commonly known as pinkeye, is a bacterial disease caused by *Moraxellabovis*. IBK is characterized by excessive tearing and ulceration of the cornea. Perforation of the cornea may also occur in severe cases. IBK is considered the most important ocular disease in cattle production, due to the decreased growth performance of infected individuals and its subsequent economic effects. IBK is an economically important, lowly heritable categorical disease trait. Mass selection of unaffected animals has not been successful at reducing disease incidence. Genome-wide studies can determine chromosomal regions associated with IBK susceptibility. The objective of the study was to detect single-nucleotide polymorphism (SNP) markers in linkage disequilibrium (LD) with genetic variants associated with IBK in American Angus cattle.

**Results:**

The proportion of phenotypic variance explained by markers was 0.06 in the whole genome analysis of IBK incidence classified as two, three or nine categories. Whole-genome analysis using any categorisation of (two, three or nine) IBK scores showed that locations on chromosomes 2, 12, 13 and 21 were associated with IBK disease. The genomic locations on chromosomes 13 and 21 overlap with QTLs associated with Bovine spongiform encephalopathy, clinical mastitis or somatic cell count.

**Conclusions:**

Results of these genome-wide analyses indicated that if the underlying genetic factors confer not only IBK susceptibility but also IBK severity, treating IBK phenotypes as a two-categorical trait can cause information loss in the genome-wide analysis. These results help our overall understanding of the genetics of IBK and have the potential to provide information for future use in breeding schemes.

## Background

Infectious Bovine Keratoconjunctivitis (IBK), commonly known as pinkeye, is a highly contagious ocular bacterial cattle disease which occurs in cattle populations throughout the world. IBK is caused by the Gram negative bacterium *Moraxella bovis*[1] and is characterized by excessive tearing, inflammation of the conjunctiva, and ulceration of the cornea in one or both eyes. As the disease progresses the cornea becomes cloudy or white. In severe cases, perforation of the cornea may occur which can lead to permanent blindness.

IBK is non-fatal; however, it is considered the most important ocular disease in cattle, due to the decreased growth performance of infected individuals. The United State National Animal Health Monitoring System survey [2] and Australian postal survey [3] reported IBK as an economically important disease in animal production. Major economic losses are the result of inappetence and poor weight gain in affected animals suffering from ocular pain and visual impairment [1]. It has been estimated that IBK costs beef producers 150 million US$ in the United States [4] and 22 million AUD$ in Australia [3] per annum.

There has been little research to quantify genetic variation in susceptibility to IBK. Breed differences in susceptibility to IBK have been demonstrated and Hereford cattle were found to be more susceptible compared with all other purebreds (Angus, Braunvieh, Charolais, Gelbvieh, Limousin, Pinzgauer, Red Poll and Simmental) and *Bos Indicus* breeds [5,6]. Heritability estimates (0.10 - 0.25) different from zero have been reported, highlighting the presence of within-breed genetic variation in susceptibility to IBK.

Putative QTL on Chromosomes 1 (66 to 110 cM) and 20 (2 to 35 cM) have been reported [7] to be associated with resistance to IBK. Reducing IBK through selection would be advantageous, because genetic gain is cumulative and permanent. Once the loci associated with susceptibility or resistance to IBK are identified, relevant markers can be used for selection in breeding schemes.

In recent years, high-density single nucleotide polymorphism (SNP) genotyping assays for cattle have become available [8]. In addition, statistical methodologies have been developed to make use of these SNP panels to detect association between SNP and economically important traits. The use of genome-wide studies helps identify chromosomal regions associated with disease incidence in immunity-related diseases such as IBK. The objective of this study was to detect SNP markers in linkage disequilibrium (LD) with genetic variants associated with IBK in Angus cattle.

## Methods

### Ethics statement

All animal procedures were approved by the Iowa State University Animal Care and Use Committee.

### Phenotypic data

Records of IBK were collected from 860 animals born and raised in the Iowa State University Angus research herd from spring 2004 through spring 2008. The IBK status of each animal was determined at weaning time and was scored subjectively into five categories for left and right eyes as follows:

(1) $$\;y_{L/R,i}=\left\{\begin{array}{ll} 1\; & \text{normal cornea with no apparent lesions},\\ 2\; & \text{a lesion covering less than 1/3 of the cornea},\\ 3\; & \text{a lesion covering 1/3 to 2/3 of the cornea},\\ 4\; & \text{a lesion covering more than 2/3 of the cornea},\\ 5\; & \text{perforation of the cornea},\\ \; \end{array}\right)$$

where *y*_*L*/*R*,*i*_ is the left (*L*) or right (*R*) eye IBK score of animal *i* and *i*=1,…,*n* (Table 1). Infection status was studied by pooling left and right eye IBK scores in various manners (Table 2).

**Table 1:** Number of animals diagnosed with different severities of Infectious Bovine Keratoconjunctivitis for left, right or both eyes

			**Right eye**			
**Left eye**	**1**	**2**	**3**	**4**	**5**	**Total**
1	539	47	26	14	16	642
2	63	23	9	6	3	104
3	30	13	10	3	1	57
4	16	7	1	4	1	29
5	15	3	3	2	5	28
Total	663	93	49	29	26	860

**Table 2:** Number of animals diagnosed with different severities of Infectious Bovine Keratoconjunctivitis classified within two, three or nine categories

	**IBK score**								
**Category**	**1**	**2**	**3**	**4**	**5**	**6**	**7**	**8**	**9**
2	539	321							
3	539	227	94						
9	539	110	79	52	54	10	8	3	5

First, left and right IBK scores were combined to form a classification involving two categories (*c*=2): Incidence was scored as 1 for both eyes unaffected and as 2 otherwise.

(2) $$y_{c=2,i}=\left\{\begin{array}{ll} 1\; & y_{L,i}=1\text{and}\;y_{R,i}=1,\\ 2\; & \text{otherwise}. \end{array}\right)$$

Second, left and right IBK scores were combined into a three category classification (*c*=3): Incidence was scored as 1 for both eyes unaffected, 2 for a single affected eye and 3 for both affected eyes.

(3) $$y_{c=3,i}=\left\{\begin{array}{ll} 1\; & y_{L,i}=1\text{and}\;y_{R,i}=1,\\ 2\; & y_{L,i}\neq 1\text{or}\;y_{R,i}\neq 1,\\ 3\; & y_{L,i}\neq 1\text{and}\;y_{R,i}\neq 1, \end{array}\right)$$

Third, left and right IBK scores were classified in nine categories (*c*=9): Incidence was scored from 1 to 9 by adding the scores of the left and right eyes (*y*_*L*,*i*_+*y*_*R*,*i*_−1).

(4) $$y_{c=9,i}=\left\{\begin{array}{l} \;1\\ \;2\\ \vdots \;y_{L,i}+y_{R,i}-1,\\ \;8\\ \;9 \end{array}\right)$$

### 50k SNP data

High-density (53,367) SNP genotypes of purebred American Angus cattle were obtained using the Bovine SNP50 Infinium II BeadChip (Illumina, Inc., San Diago CA). The Illumina A/B allele calls were used to compute a covariate for each locus that had values 0, 1, or 2 to represent the number of B alleles. Missing genotypes represented less than 0.2% of total observations and were replaced with average covariate values. All genotypes were retained in the analysis regardless of minor allele frequency.

### BayesB threshold model

The threshold model for ordered categorical data assumes the existence of a set of ordered thresholds *τ*_0_=−*∞*<*τ*_1_<*τ*_2_<…<*τ*_*c*−1_<*τ*_*c*_=*∞* for the trait and an underlying or latent variable *l*_*i*_ for each animal *i*. When the latent variable for animal *i* is between thresholds *τ*_*j*−1_ and *τ*_*j*_ the categorical phenotype *y*_*i*_ for animal *i* takes value *j*[9,10].

The BayesB [11] model for genome-wide analysis of continuous traits has previously been extended [12] to analyze categorical phenotypes assuming a threshold model as described below. In this approach, the latent variable *l*_*i*_ corresponding to *y*_*i*_, the IBK categorical phenotype, is modeled as follows:

(5) $$l_{i}={\text{x}}_{i}^{\prime }\beta +\overset{K}{\underset{k=1}{\sum }}z_{\text{ik}}\alpha_{k}+e_{i},\;i=1,\mathrm{.....},n$$

where ***β*** is a *p*x1 vector of fixed effects, ${\text{x}}_{i}^{\prime }$ is a known incidence row vector corresponding to fixed effects in ***β***, *K* is the number of marker loci, *z*_*ik*_ is the value of the covariate for marker *k* in individual *i*, *α*_*k*_ is the random substitution effect for locus *k*, with $\alpha_{k}∼N(0,\sigma_{\alpha_{k}}^{2})$ with probability 1- *π* or *α*_*k*_=0 with probability *π*. For a locus included in the model, the locus-specific variance, $\sigma_{\alpha_{k}}^{2}$, is assumed to have a scaled inverse chi-square distribution with degrees of freedom *ν*_*α*_=4 and scale parameter $S_{\alpha }^{2}=\frac{\sigma_{g}^{2}(\nu_{\alpha }-2)}{(1-\pi )\overset{K}{\underset{k=1}{\sum }}2p_{k}(1-p_{k})\nu_{\alpha }}$, where *p*_*k*_ is the B allele frequency of marker *k* and $\sigma_{g}^{2}$ is the additive genetic variance explained by markers [13,14]. For a locus not in the model, $\sigma_{\alpha_{k}}^{2}$ is set to zero. A flat prior was assumed for the fixed effects and thresholds, and the residual, *e*_*i*_, was assumed to be distributed $N(0,\sigma_{e}^{2}=1)$.

The joint posterior density of ***β***,***α***,***τ***, $\sigma_{\alpha }^{2}$ and ***l***[10] is given by:

(6) $$\;p(\beta ,\alpha ,l,\tau ,\sigma_{\alpha }^{2}\;|y)=\overset{n}{\underset{i=1}{\prod }}\left[\mathrm{Pr}(y_{i}\;=\;j|l_{i},\tau )p(l_{i}|\beta ,\alpha )\right]p(\beta ,\alpha ,\tau ,\sigma_{\alpha }^{2})$$

where $\alpha ,\sigma_{\alpha }^{2},\tau$ and ***l*** are the vectors of marker effects, marker variances, thresholds and liabilities.

Given the threshold model, the conditional probability Pr(*y*_*i*_=*j*|*l*_*i*_,***τ***) in equation (6) is either 1 or zero, and following Albert and Chib [15] this probability can be written as:

(7) $$\mathrm{Pr}(y_{i}=j|l_{i},\tau )=\overset{c}{\underset{j=1}{\sum }}I(\tau_{j-1}<l_{i}<\tau_{j})I(y_{i}=j),$$

where *I*(.) is an indicator function taking the value 1 when expression (.) is true and 0 otherwise.

It is assumed that, given the location parameters ***β*** and ***α***, the density function of the latent variable *l*_*i*_ in equation (6) is normal:

(8) $$p(l_{i}|\beta ,\alpha )=N({\text{x}}_{i}^{\prime }\beta +\overset{K}{\underset{k=1}{\sum }}z_{\text{ik}}\alpha_{k},1).$$

The density of the joint prior distribution in equation (6) has the form

(9) $$\begin{array}{c} \begin{array}{c} p(\beta ,\alpha ,\tau ,\sigma_{\alpha }^{2})=p(\beta )p(\alpha )p(\tau )p(\sigma_{\alpha }^{2}). \end{array} \end{array}$$

A Gibbs sampler was used to construct an irreducible, Markov chain with the joint posterior as its stationary distribution [10,16,17]. Inferences were made from the Markov chain. In the Gibbs sampler, samples are obtained from the full conditional posteriors rather than the joint posterior [10,16,17]. The full conditional posteriors used in this paper are described below.

The full conditional posterior for the fixed effect *β*_*m*_ given all other unknowns is a normal distribution:

(10) $$p(\beta_{m}|\beta_{-m},\alpha )=N({\hat{\beta }}_{m},{\left({\text{x}}_{m}^{\prime }{\text{x}}_{m}\right)}^{-1})$$

where

(11) $${\hat{\beta }}_{m}=\frac{{\text{x}}_{m}^{\prime }(l-[{\text{X}}_{-m}\beta_{-m}+\overset{K}{\underset{k=1}{\sum }}z_{k}\alpha_{k}])}{{\text{x}}_{m}^{\prime }{\text{x}}_{m}},$$

**X**_−*m*_ is the matrix **X** with the column associated with *m* deleted, and ***β***_−*m*_ is ***β*** with the *m*th element deleted.

At locus *k*, following Meuwissen *et al*[11], the locus-specific marker variance, $\sigma_{\alpha_{k}}^{2}$, and the SNP effect, *α*_*k*_, were sampled from their joint full conditional posterior distribution. The strategy they used was to sample $\sigma_{\alpha_{k}}^{2}$ from the marginal of the full conditional posterior and then sample *α*_*k*_ from the conditional posterior given the sampled value for $\sigma_{\alpha_{k}}^{2}$[11].

This full-conditional distribution does not have a closed form, and thus the Metropolis-Hasting (MH) algorithm is used to sample $\sigma_{\alpha_{k}}^{2}$. Meuwissen *et al*[11] used the prior distribution of $\sigma_{\alpha_{k}}^{2}$ as the proposal distribution in the MH algorithm. Here we used the proposal distribution described by Habier *etal*. [14]. Given the sampled value of $\sigma_{\alpha_{k}}^{2}$, the marker effect *α*_*k*_ was sampled from its conditional posterior:

(12) $$\alpha_{k}|\sigma_{\alpha_{k}}^{2}=\left\{\begin{array}{ll} 0 & \sigma_{\alpha_{k}}^{2}=0,\\ ∼N(\frac{{\text{z}}_{k}^{\prime }r_{k}}{C_{k}},C_{k}^{-1}) & \sigma_{\alpha_{k}}^{2}>0, \end{array}\right)$$

where $r_{k}=l-[\text{X}\beta +\overset{K}{\underset{k^{\prime }\neq k}{\sum }}z_{k^{\prime }}\alpha_{k^{\prime }}]$ and $C_{k}=z_{k}^{\prime }z_{k}+{\left(\sigma_{\alpha_{k}}^{2}\right)}^{-1}$[14].

Following Cowles [18], the latent variables and thresholds were sampled jointly from their joint full conditional distribution. Cowles [18] samples the thresholds from the marginal of this full conditional posterior, which is obtained by integrating out the latent variables [19]. The marginal full conditional does not have a closed form and an MH algorithm is used to draw samples of the thresholds [18]. Then, given the sampled values of the thresholds, each of the latent variables is sampled from its conditional posterior, which is truncated normal:

(13) $$\begin{array}{c} p(l_{i}|\beta ,\alpha ,\tau ,l_{-i},y)\propto N({\text{x}}_{i}^{\prime }\beta +\overset{K}{\underset{j=1}{\sum }}z_{\text{ik}}\alpha_{k},1)\\ \;\times \left(\overset{c}{\underset{j=1}{\sum }}I(\tau_{j-1}<l_{i}<\tau_{j})I(y_{i}=j)\right), \end{array}$$

as described by Devroye [20].

### Genetic variance and heritability

The posterior means of the genetic variance and of the heritability for the latent variable were estimated from the Markov Chain Monte Carlo (MCMC) samples as follows. In each MCMC step, the vector **g** of breeding values of all animals was sampled as:

(14) $${\text{g}}^{\left(t\right)}=\overset{K}{\underset{k=1}{\sum }}z_{k}\alpha_{k}^{\left(t\right)},$$

where $\alpha_{k}^{\left(t\right)}$ is the sampled value of *α*_*k*_ in step *t*. Now, vector **g**^**(t)**^ was used to sample the genetic variance $\sigma_{g}^{2(t)}$ as

(15) $$\sigma_{g}^{2(t)}=\frac{\overset{n}{\underset{i}{\sum }}{\left(g_{i}^{\left(t\right)}-{\bar{g}}^{\left(t\right)}\right)}^{2}}{n},$$

where

$${\bar{g}}^{\left(t\right)}=\frac{\overset{n}{\underset{i}{\sum }}g_{i}^{\left(t\right)}}{n}.$$

The heritability *h*^2(*t*)^ was then sampled as

(16) $$h^{2(t)}=\frac{\sigma_{g}^{2(t)}}{\sigma_{g}^{2(t)}+1}.$$

The arithmetic means of these samples were used to estimate their posterior means.

### Window variance

The genetic variance attributed to a genomic window is defined as follows. First, the breeding value for a genomic window is defined as

(17) $${\text{g}}_{w}=\underset{k\in W}{\sum }z_{k}\alpha_{k},$$

where *α*_*k*_ is the effect of SNP *k* and *W* is the set of indices of SNPs that belong to genomic window *w*. The genetic variance attributed for genomic window *w* is now defined as

(18) $$\sigma_{g_{w}}^{2}=\frac{\overset{n}{\underset{i}{\sum }}{\left(g_{w_{i}}-{\bar{g}}_{w}\right)}^{2}}{n},$$

where

$${\bar{g}}_{w}=\frac{\overset{n}{\underset{i}{\sum }}g_{w_{i}}}{n}.$$

Samples of window variances were obtained using (15) but with **g**^(*t*)^ computed with only the sampled SNP effects for window *w*[21]. These samples were used to compute posterior means and posterior probabilities for percent window variances and were used for inferences on locations of causal variants.

### Bioinformatics and identification of candidate genes

SNP effects from genome-wide analysis were obtained using the threshold model in GenSel software [22] and the estimated effects were uploaded into SNPLOTz (http://www.animalgenome.org/tools/snplotz). SNPLOTz visualized the estimated SNP effects and their genomic location and was dynamically linked to Gbrowse (http://www.animalgenome.org/gbrowse), which allowed visualization of SNP with other types of genome features (i.e., annotated genes, curated QTL, transcripts, etc.) [23]. The SNP positions within a chromosome were based on *Bos taurus* genome assembly (UMD 3.1).

## Results and discussion

The distribution of IBK records by left and right eyes are presented in Table 1. The rates for severity of IBK that was scored into five categories from cornea with no apparent lesions to perforation of the cornea were 75, 12, 7, 3 and 3% for left eye and 77, 11, 6, 3 and 3% for right eye. These results indicated similarity between left and right eyes for IBK incidence rates. Rodriguez [24] studied the effects of IBK incidence severity on production traits in Angus breed and found similar incidence rates between left and right eyes.

The distribution of IBK scores classified in two, three or nine categories of infection can be seen in Table 2. The rates of unaffected, affected, only one-eye affected and two-eye affected animals were 63, 37, 26 and 11%, respectively. The severity score of IBK within the nine category classification system varied from 12.8% to 0.58% for infected animals. These results were found to be similar with those from Rodriguez [24]; however, higher than those (3.7%) from Snowder *et al.*[6], that studied environmental effects and genetic factors influencing the incidence of IBK among nine breeds including Angus. A significant between-breed difference in IBK incidence has been reported [25], with purebred Herefords (22.4%) being more susceptible compared to other pure (Simmental 7.6%, Charolais 6.5%, Angus 3.7%, Limousin 3.4%) and composite breeds. Thus, sufficient genetic variation for resistance to IBK exists in these breeds to consider selection.

### Inference on heritability

**Figure 1:**
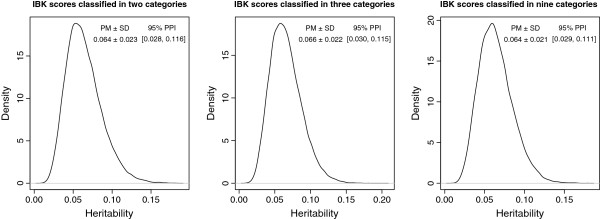
**Posterior inference on heritability of Infectious Bovine Keratoconjunctivitis scores classified within two, three or nine categories in American Angus cattle.PM:** Posterior mean, **SD:** Posterior standard deviation, **PPI:** 95% posterior probability interval.

The posterior distribution of heritability for each of the IBK scores classified into two, three or nine categories is shown in Figure 1, along with the posterior mean (PM), posterior standard deviation and 95% posterior probability interval (PPI), defined to be the range of posterior density falling between the 2.5th and 97.5th percentile.

The posterior distribution of heritabilities seemed similar across the different classification of IBK scores. Posterior means of heritabilities for two- (0.064) and nine-category (0.064) IBK scores were the same; however, they were slightly lower than 0.066 for three-category IBK scores. The 95% PPI of heritabilities overlapped, which indicates that there is no significant difference among heritability estimates of IBK incidence based on different classifications.

There are very few studies reporting estimates of heritability for IBK incidence in the literature. Our heritability estimates were lower, but in good agreement with the estimates (0.06 - 0.10) obtained from Rodriguez [24]. However, our heritability estimates are lower than the heritability estimates of 0.10-0.25 obtained by Snowder *et al.*[6] based on the models including only animal effect, and animal and maternal effects for Angus cattle. In the same study, Snowder *et al.*[6] also found high heritability estimates (0.20-0.28) for Hereford and low heritability (0.0 - 0.13) estimates for Red Poll, Charolais, Simmental, Limousin, Gelbvieh, Pinzgauer, Braunvieh breeds. Ali *et al.*[26] indicated that estimates of heritability for IBK were small to moderate (0.17 - 0.19) for both pre-weaning and post-weaning calves in a Hereford and Shorthorn composite population. These differences between heritability estimates could be attributed partly by the differences in phenotypes measured, failure to accurately distinguish between phenotypes of healthy and sick animals; false assumption that disease observed is the primary infection, lack of knowledge of the influence of passive immunity on disease incidence, susceptibility of disease biased by time, age, or season dependency and/or differences in the models of analyses used in these studies [6]*,*[24].

### Effect of different categorisation of infectious bovine keratoconjunctivitis on the whole-genome analysis

**Figure 2:**
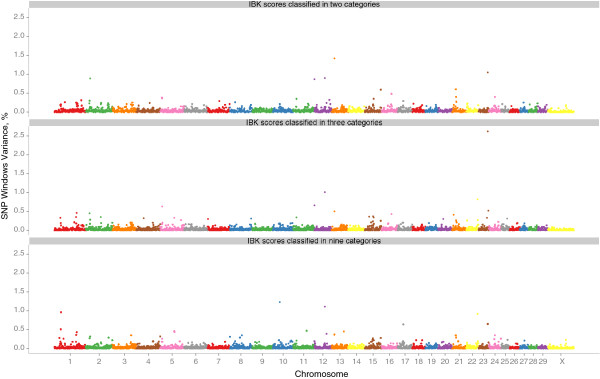
**Plot of the proportion of window variance accounted for by genome locations for two, three or nine IBK scores in American Angus cattle.** Each spot on the plot indicates the proportion of genetic variance contributed by a SNP window defined based on *Bos taurus* genome assembly (UMD 3.1). The colors represent SNP windows from chromosome 1 to X.

Genome-wide Manhattan plots that display the proportion of genetic variance explained by all 2,648 1-Mb SNP windows with respect to their genomic positions are shown for IBK scores classified as two, three or nine categories in Figure 2. Additional Table S1, S2 and S3 in the Additional file 1 also present the information about the top thirty 1-Mb SNP windows explaining 0.20% or more of the genetic variance observed in IBK classifications. The top thirty 1-Mb SNP windows explained 13.3, 14.5 and 14.0% of genetic variance observed in IBK scores classified as two, three or nine categories, respectively. As seen from Figure 2 and Tables in the Additional file 1, whole-genome analysis for each categorisation (two, three or nine) of IBK scores indicated that similar genomic regions were associated with IBK incidence although the variance explained by each region differed slightly according to the categorisation used.

Two-category classification of IBK was performed by considering all animals whose IBK status was at least as severe as the threshold level to be ‘affected’, while considering all animals whose IBK status was less severe than the threshold level to be ‘unaffected’. It was also assumed that animals with IBK had the same levels of exposure to the genetic risk factors. In the two-category classification, however, some animals considered ‘affected’ could be in more severe states than other ‘affected’ animals [27]. This additional information could be used in the multiple-category classification of IBK, whereas it was lost upon two-category classification. In the linkage analyses of two-, three- and multiple-categorical traits, Corbet *el al.*[27] indicated that two-category classification of multiple-categorical traits could lead to crippling power loss, especially in the case of many loci of small effects. In particular, if the underlying genetic factors for any trait confer not only IBK susceptibility but also IBK severity, treating IBK phenotypes as multi-categorical could provide additional information in genome-wide analysis.

### Association analysis for infectious bovine keratoconjunctivitis

Figure 2 and Tables in the Additional file 1 indicated many genomic regions with different degree of association with IBK incidence in American Angus breed. Casas and Stone [7] had previously reported presence of QTL for IBK tolerance on chromosome 1 and 20. Casas and Snowder [25] further indicated that the region on chromosome 20 might be associated with general resistance to bacterial diseases (including IBK). In this present study, several SNP windows associated with IBK disease status were identified on chromosome 1 (Additional file 1). However, three SNP windows ([109.01 Mb] - [109.87 Mb], [110.04 Mb] - [110.98 Mb] and [112.04 Mb] - [113.00 Mb]) on chromosome 1 overlapped previously described QTL region for IBK susceptibility [7]. There are two annotated genes within these 1-Mb SNP windows on chromosome 1; *PTX3* (pentraxin 3) and *IL12A* (interleukin 12A, natural killer cell stimulatory factor 1, cytotoxic lymphocyte maturation factor 1, p35).

*PTX3* has been reported to be rapidly produced and released by several cell types, in particular by mononuclear phagocytes, dendritic cells, fibroblasts and endothelial cells [28]*,*[29] and recognizes microbial moieties, opsonizes fungi and selected Gram-positive and Gram-negative bacteria and activates complement. Opsonization resulted in facilitated pathogen recognition and innate immune cell activation; moreover, opsonization by *PTX3* is likely to be involved in the activation of an appropriate adaptive immune response [29]. *IL12A*, an immunomodulatory cytokine secreted by antigen presenting cells, is critical for differentiation of T helper (Th)1 and Th2 lymphocytes [30]. *IL12* has been shown to augment the growth of activated T- and natural killer (NK)-cells [31], stimulate interferon gamma (IFN- *γ*) production by T-cells and NK cells, and suppress the expansion of Th2 cell clones [32]. This information about *PTX3* and *IL12A* genes indicates that they could be strong biological as well as positional candidates involved in response to IBK infections.

It is important to note that in the present study there were no common 1-Mb SNP windows on chromosome 20 for IBK incidence. While this result was not consistent with the results from the previous studies [7]*,*[25] about IBK incidence, the difference may be in part due to design differences between this study (a single breed of purebred *Bos taurus* animals) and the previous studies (a single *Bos taurus* x *Bos indicus* sire bred to multiple breeds of cows). These differences in results may indicate different metabolic pathways or different segregating QTL (within breed vs. across breed) for IBK incidence.

In the present work, five 1-Mb SNP windows (Table 3), which were identified as being associated with IBK incidence, were determined as common windows within the thirty SNP windows for IBK classifications (Tables in Additional file 1): *rs109448194* - *rs42270183* and *rs41642303* - *rs110857971* on chromosome 2, *rs108956311* - *rs43705367* on chromosome 12, *rs29021773* - *rs109429649* on chromosome 13 and *rs41966737* - *rs41640647* on chromosome 21. The percentage of genetic variance explained by these five 1-Mb SNP windows based on two-, three- and nine-category of IBK incidence were 0.30, 0.45 and 0.27%, and 0.89, 0.28 and 0.32% for two SNP windows on chromosome 2; 0.90, 1.01 and 1.11% for SNP window on chromosome 12; 1.42, 0.50 and 0.37% for SNP window on chromosome 13 and 0.40, 0.26 and 0.29% for SNP window on chromosome 21, respectively. These results showed that same genomic regions could have different degree of association with IBK incidence based on the two-, three-, or nine-category classification of IBK incidence, which means IBK could have separate sets of genes controlling affection and severity of the incidence.

**Table 3:** Common 1-Mb SNP windows from the analyses of IBK incidence across two, three and nine categories

		**rs number of**	**Position of**	**rs number of**	** Position of**	**Number of**	**Proportion of genetic variance in IBK classified in**		
**Chromosome**	**Mb**	**first SNP**	** first SNP**	**last SNP**	** last SNP**	**SNP in window**	**Two categories**	**Three categories**	**Nine categories**
2	19	rs109448194	19105629	rs42270183	19965497	19	0.30	0.45	0.27
2	22	rs41642303	22087692	rs110857971	22768217	14	0.89	0.28	0.32
12	53	rs108956311	53009331	rs43705367	53986983	23	0.90	1.01	1.11
13	14	rs29021773	14356314	rs109429649	14923596	11	1.42	0.50	0.37
21	19	rs41966737	19002483	rs41640647	19983053	27	0.40	0.26	0.29

The SNP loci were based on *Bos taurus* genome assembly (UMD 3.1).

Several candidate genes in the five 1-Mb SNP windows were identified and listed with their functions in Table 4. Examining the first 1-Mb SNP window [19.10 - 19.97 Mb] on chromosome 2 indicated two genes; *AGPS* (alkylglycerone phosphate synthase), *NFE2L2* (nuclear factor (erythroid-derived 2)-like 2). *AGPS* is a peroxisomal enzyme which is required for the synthesis of plasmalogens. Liegel *et al.*[33] indicated that a mutation based on a G to A substitution at the +5 position of intron 14 in the *AGPS* gene resulted in severe plasmalogen deficiency which is the cause of cataracts and male sterility in the blind sterile 2 mice having spontaneous autosomal recessive mutation. *NFE2L2* (also called *NRF2*) is a basic leucine zipper transcription factor that mediates the cytoprotective cellular antioxidant response [34]. Wangy *et al.* indicated that *NFE2L2* was an essential transcription factor in protecting living organisms from oxidative stress-related disease. Ungvari *et al.*[35] showed that polymorphisms in the regulatory regions of *NFE2L2* are associated with susceptibility to infection-induced asthma in the study of relationship between air pollution, *NFE2L2* gene polymorphisms and childhood asthma in a Hungarian population. They also found remarkable differences in the genotype distributions of these polymorphisms between different polluted regions, which indicate an environment-dependent regulation of the antioxidant defense mechanisms. This *NFE2L2* gene could be associated with susceptibility to IBK incidence because several factors including the environment, season, fly concentration, the presence of the pathogen, strain of the pathogen and background of the animal play a role in the incidence, penetrance, and severity of IBK [36]. In addition, Schwalfenber [37] reported the role of vitamin D in the prevention of viral, fungal and bacterial (such as ocular) infections. He indicated that 1,25(OH)2D, active form of vitamin D, has been shown to increase the induction of genes encoding for human *β* defensin which promotes resistance to eye infection by *P. aeruginosa*. Epidemiological studies have linked vitamin D deficiency to increased rates of cancer, as well as autoimmune and infectious diseases [37]. Vitamin D-mediated protection from pro-oxidant stress was determined to be indirect due to the induction of *NFE2L2*[38] and it was found that *NFE2L2* expression was down-regulated in prostate cancer and suppression of *NFE2L2* promotes prostate tumor development in TRAMP mice [39].

**Table 4:** Positional candidate genes (PCG) within 1-Mb SNP window (SNPW) and their putative functions

**Chromosome**	**SNPW**	**PCG**	**Gene ontology**
2	19.11 - 19.97	AGPS	It encodes a protein that catalyzes the step of lipid biosynthesis
			and the removal of long chain acid anion
		NFE2L2	It encodes a transcription factor which is a member of a small
			family of basic Leucine (bZIP) proteins
2	22.09 - 22.77	WIPF1	It encodes a protein that plays an important role in the organization
			of the actin cytoskeleton
		OLA1	It hydrolyzes ATP and can hydrolyze GTP with lower efficiency
		SP3	regulates transcription by binding consensus GC and GT- box
			elements in target genes
12	53.01 - 53.99	SCEL	It encodes a tissue-specific basic helix-loop-helix (bHLH) protein
			with a pivotal role in hemopoises
		EDNRB	it mediates actin by association with G-Protein that activates
			a phosphaidylinositol-calcium second messenger system
13	14.36 - 14.92	VAMP7	It encodes a transmembrane protein that is a member of soluble
			N-ethylmeleimide-sensitive factor attachment protein receptor
			(SNARE) family
21	19.00 - 19.98	NTRK3	It encodes a member of a neurotrophic tyrosine receptor kinase family
		MIR1179	It involved in post transcriptional regulation of gene expression
		MIR7-1	It involved in post transcriptional regulation of gene expression

The second SNP window [22.09 - 22.77 Mb] harbors 3 genes; *WIPF1* (WAS/WASL interacting protein family, member 1), *OLA1* (Obg-like ATPase 1), *SP3* (Sp3 transcription factor). *WIPF1* encodes a protein that plays an important role in the organization of the actin cytoskeleton. Wickramarachchi *et al.*[40] indicated that deficiency of actin regulators can result in defects limited to the immune system or sometimes to a single immune cell type and lack of an actin cytoskeletal regulator can cause immunodeficiency, autoimmunity, autoinflammatory disease, or a combination of these manifestations. In addition, Dustin and Cooper [41] pointed out that the actin cytoskeleton seems to play two critical roles in the activation of T cells: T cell shape development and movement, including formation of the immunological synapse and the formation of a scaffold for signaling components. *OLA1* is a negative regulator of the antioxidative process. Knockdown of *OLA1* in human cells elicited an increased resistance to oxidizing agents. Zhanga *et al.*[42] also reported that knockdown of *OLA1*, a newly discovered regulatory protein of oxidative stress response, inhibits cell migration and invasion ability in breast cancer cells. *Sp* factors are able to stimulate transcription from proximal promoters or from distal enhancers. They can also physically interact with other transcription factors. van Loo *et al.*[43] studied the impact of the absence of the widely expressed transcription factor *SP3* on the developing hematopoietic system in the mouse. They showed that the absence of *SP3* results in cell-autonomous differentiation defects in the erythroid and myeloid cell lineage. *WIPF1*, *OLA1*and *SP3* genes are found to be associated with defects in immune system or cell lineage making them biological candidate for IBK disease.

Two genes were identified within the SNP window [53.01 - 53.99 Mb] on chromosome 12; *SCEL* (sciellin), *EDNRB* (endothelin receptor type B). Sciellin is a protein that is encoded by the *SCEL* gene. it is a precursor of the cornified enveloped. The cornified envelope is an insoluble protein complex formed under the plasma membrane in the uppermost layers of stratified squamous epithelium and plays a major role in the barrier properties of the stratum corneum [44]. Champliaud *et al.*[45] reported proteins interacting with sciellin and identified vitamin D-upregulated protein 1 (VDUP1), which plays multiple roles in a wide range of cellular processes such as proliferation or apoptosis. Recently, it has been reported that VDUP1 is also involved in the immune system via positive regulation of natural killer development [46]. The endothelin receptor B (*EDNRB*) gene encodes a g-protein-coupled receptor-mediated endothelin, inducing development and transformation of the neural crest cell-specific lineage. Hypermethylation of the *EDNRB* promotor has been shown in multiple tumor types, and *EDNRB* has been proposed as a putative tumor suppressor gene [47].

One gene is located within SNP window [14.36 - 14.92 Mb] on chromosome 13; *VAMP7* (vesicle-associated membrane protein 7). *VAMP7* inhibits the release of lytic granules and severely impairs natural killer (NK) cell cytotoxic activity which is used to eliminate cancer and virus-infected cells. Furthermore, *VAMP7* is involved in IFN secretion in NK cells, which indicates that *VAMP7* is involved in many fusion processes and thus plays a general function in NK cell activity [48]. In addition, this SNP window lies in the region where putative QTLs that affect Bovine spongiform encephalopathy at 22.997-61.84 cM resides [49]. These findings indicate that these genes could be candidates for IBK susceptibility based on their functions.

The SNP window [19.00 - 19.98 Mb] on chromosome 21 harbors three genes that encode proteins; *NTRK3* (neurotrophic tyrosine kinase, receptor, type 3), *MIR1179* (microRNA mir-1179), *MIR7-1* (microRNA mir-7-1). *NTRK3* gene encodes a member of the *NTRK* family. These neurotrophins (NTs) receptors are best known for their role in the differentiation and survival of various types of neurons [50]. In the study of latitude-driven adaptation for both schizophrenia and vitamin D related genes, Amato *et al.* found 9 genes including *NTRK3* in common among those ones related to latitude, vitamin D and schizophrenia. MicroRNAs (miRNAs) are small 21-23 nucleotide-long noncoding RNAs involved in several biological process including development, differentiation, apoptosis, survival, motility, invasion and proliferation. Many miRNAs are implicated as proto-oncogenes or as tumor suppressors and aberrantly expressed in various cancer types. MIR7 has been characterized as a tumor suppressor in several human cancers. This SNP window also lies in the region where putative QTLs that affect clinical mastitis at 12.60-35.89 cM and somatic cell scores at 12.601-29.77 cM, have been identified by Schulman *et al.*[51] and Schnabel *et al.*[52], respectively. These results indicate that genetic variant(s) that reside in this chromosomal region could be associated with bacterial disease such as IBK in cattle.

## Conclusions

The present analyses, using Bovine SNP50 Infinium II BeadChips, identified several 1-Mb SNP regions and genes within these regions that were associated with IBK. They may be of interest for breeding schemes as they could be used to identify animals susceptible to IBK.

IBK disease is a lowly heritable complex trait that is polygenic in nature, where many loci with small effects are expected and disease incidence is affected by the environment. Such traits require a large number of individuals to enhance the power of the genome-wide analysis and obtain good statistical support for the detection of the causal loci.

## Competing interests

The authors declare that they have no competing interests.

## Authors’ contributions

KK developed the model and part of the program, and carried out the statistical analysis. RGT and JMR did the data preparation and helped with the statistical analysis. DJG and RLF developed the GenSel program used for analysis. KK, RGT, DJG, RLF and JMR helped to draft the manuscript. All authors read and approved the final manuscript.

## Supplementary Material

Additional file 1 Top thirty 1-Mb SNP windows from genome-wide association study of IBK classified in two, three or nine categories. Click here for file

## References

[B1] McConnel CS Shum L House JK Infectious bovine keratoconjunctivitis antimicrobial therapy Aust Vet J 2007 85 65 69 10.1111/j.1751-0813.2006.00080.x 17300463

[B2] NAHMS Part III. Reference of 1997, beef cow-calf production management and disease control USDA,APHIS, Natl Anim Health Monit Syst 1998 [http://www.aphis.usda.gov/animal_health/nahms/beefcowcalf/downloads/beef97/Beef97_dr_PartIII.pdf]

[B3] Slatter D Edwards M Hawkins C A national survey of the accurrence of infectious bovine keratoconjunctivitis Aust Vet J 1982 59 65 68 10.1111/j.1751-0813.1982.tb02728.x 7159308

[B4] Hansen R New tools in the battle against pinkeye Nevada Livest. Prod. Annu. Update 2001 Univ. of Nevada–Reno, UNR Coop. Ext. SP 01–01 5 8

[B5] Frisch JE The relative incidence and effect of bovine infectious keratoconjunctivitis in bos indicus and bos taurus cattle Anim Prod 1975 21 265 274 10.1017/S0003356100030737

[B6] Snowder GD Van Vleck LD Cundiff LV Bennett GL Genetic and environmental factors associated with incidence of infectious bovine keratoconjunctivitis in preweaned beef calves J Anim Sci 2005 83 507 518 15705746 10.2527/2005.833507x

[B7] Casas E Stone RT Putative quantitative trait loci associated with the probability of contracting infectious bovine keratoconjunctivitis J Anim Sci 2006 84 3180 3184 10.2527/jas.2006-200 17093209

[B8] Matukumalli LK Lawley CT Schnabel RD Taylor JF Allan MF Heaton MP O’Connell J Moore SS Smith TP Sonstegard TS Van Tassell CP Development and characterization of a high density SNP genotyping assay for cattle PLoS One 2009 4 4 e5350 10.1371/journal.pone.0005350 19390634 PMC2669730

[B9] Wright S An analysis of variability in number of digits in an inbred strain of guinea pigs Genetics 1934 19 506 536 17246735 10.1093/genetics/19.6.506 PMC1208511

[B10] Sorensen DA Gianola D Likelihood, Bayesian and MCMC methods in Quantitative Genetics 2002 New York: Springer-Verlag, New York, Inc;

[B11] Meuwissen THE Hayes BJ E GM Prediction of total genetic value using genome wide dense marker maps Genetics 2001 157 1819 1829 11290733 10.1093/genetics/157.4.1819 PMC1461589

[B12] Villanueva B Fernandez J Garcia-Cortes LA Varona L Daetwyler HD Toro MA Accuracy of genome-wide evaluation for diease resistance in aquaculture breeding programs J Anim Sci 2011 89 3433 3442 10.2527/jas.2010-3814 21742941

[B13] Fernando RL Habier D Sticker C Dekkers JCM Totir LR Genomic selection Acta Agriculturae Scand 2007 57 192 195

[B14] Habier D Fernando RL Kizilkaya K J GD Extension of the Bayesian alphabet for genomic selection BMC Bioinformatics 2011 12 186 1 12 21605355 10.1186/1471-2105-12-186 PMC3144464

[B15] Albert J Chib S Bayesian analysis of binary and polychotomous response data J Am Stat Assoc 1993 88 669 679 10.1080/01621459.1993.10476321

[B16] Geman D Geman S Stochastic relaxation, Gibbs distributions, and the Bayesian restoration of images IEEE Trans Pattern Anal Intel 1984 6 721 741 10.1109/tpami.1984.4767596 22499653

[B17] Gelfand AE Smith AFM Sampling-based approaches to calculating marginal densities J Amer Stat Ass 1990 85 398 409 10.1080/01621459.1990.10476213

[B18] Cowles MK Accelerating Monte Carlo Markov Chain convergence for cumu-lative link generalized linear models Stat Comp 1996 6 101 111 10.1007/BF00162520

[B19] Chen MH Shao QM Ibrahim JG Monte Carlo Methods in Bayesian Computation 2000 New York: Springer-Verlag;

[B20] Devroye L Non-Uniform Random Variate Generation 1986 New York: Springer-Verlag;

[B21] Fernando RL Garrick D Bayesian methods applied to GWAS Genome-Wide Association Studies and Genomic Prediction 2013

[B22] Fernando R Garrick D GenSel-User manual for a portfolio of genomic selection related analyses [http://taurus.ansci.iastate.edu]

[B23] Hu Z Fernando R Garrick DJ Reecy JM SNPLOTz: a generic genome plot tool to aid the SNP association studies BMC Bioinformatics 2010 Suppl4 P4

[B24] Rodríguez JE Infectious Bovine Keratoconjunctivitis in Angus cattle Master’s thesis 200623728480

[B25] Casas E Snowder GD Putative quantitative trait loci associated with bovine pathogenic disease incidence J Anim Sci 2008 86 2455 2460 10.2527/jas.2008-0933 18502878

[B26] Ali AA Thomson PC J OC Genetic parameters of infectious bovine keratoconjunctivitis and its relationship with weight and parasite infestations in Australian tropical Bos taurus cattle Genet Sel Evol 2012 44 22 10.1186/1297-9686-44-22 PMC3517348 22839739

[B27] Corbet J Gu C Rice J Reich T Province M Rao D Power loss for linkage analysis due to the dichotomization of trichotomous phenotypes Hum Hered 2004 57 21 27 10.1159/000077386 15133309

[B28] Garlanda C Bottazzi B Bastone A Mantovani A Pentraxins at the crossroads between innate immunity, imflammation, matrix deposition, and female fertility Annu Rev Immunol 2005 23 337 366 10.1146/annurev.immunol.23.021704.115756 15771574

[B29] Battazzi B Garlanda C Salvatori G Jeannin P Manfredi A Mantovani A Pentraxins as a key component of innate immunity Current Opinion in Immunology 2006 18 10 15 10.1016/j.coi.2005.11.009 16343883

[B30] Szabo SJ Jacobson NG Dighe AS Gubler U Murphy KM Developmental commitment to the Th2 lineage by extinction of IL-12 signaling Immunity 1995 2 665 675 10.1016/1074-7613(95)90011-X 7796298

[B31] Robertson MJ Soiffer RJ Wolf SF Manley TJ Donahue C Young D Herrmann SH Ritz J Response of human natural killer (NK) cells to NK cell stimulatory factor (NKSF): cytolytic activity and proliferation of NK cells are differentially regulated by NKSF J Exp Med 1992 175 779 788 10.1084/jem.175.3.779 1346796 PMC2119162

[B32] Chung F Anti-inflammatory cytokines in asthma and allergy interleukin-10, interleukin-12, interferon-gamma Mediators Inflamm 2001 10 51 59 10.1080/09629350120054518 11405550 PMC1781697

[B33] Liegel R Chang B Dubielzig R Sidjanin D Blind sterile 2 (bs2), a hypomorphic mutation in Agps, results in cataracts and male sterility in mice Mol Genet Metab 2011 103 51 59 10.1016/j.ymgme.2011.02.002 21353609 PMC3081956

[B34] Giudice A Montella M Activation of the Nrf2–ARE signaling pathway a promising strategy in cancer prevention Bioessays 2006 28 169 181 10.1002/bies.20359 16435293

[B35] Ungvari I Hadadi E Virag V Nagy A Kiss A Kalmar A Zsigmond G Semsei AF Falus A Szalai C Relationship between air pollution, NFE2L2 gene polymorphisms and childhood asthma in a Hungarian population J Community Genet 2012 3 25 22 10.1007/s12687-011-0075-8 22207565 PMC3266964

[B36] Brown M Brightman A Fenwick B Rider M Infectious bovine keratoconjunctivitis: A review J Vet Med 1998 12 259 266 10.1111/j.1939-1676.1998.tb02120.x 9686385

[B37] Schwalfenberg G Not enough vitamin D for Canadians Can Fam Physician 2007 53 841 854 17872747 PMC1949171

[B38] Wild A Moinova H RT M Regulation of gamma-glutamylcysteine synthetase subunit gene expression by the transcription factor Nrf2 J Biol Chem 1999 274 33627 33636 10.1074/jbc.274.47.33627 10559251

[B39] Frohlich D Mccabe M Arnold R Day M The role of Nrf2 in increased reactive oxygen species and DNA damage in prostate tumorigenesis Oncogene 2008 27 4353 4362 10.1038/onc.2008.79 18372916

[B40] Wickramarachchi D Theofilopoulos A Kono D Immune pathology associated with altered actin cytoskeleton regulation Autoimmunity 2010 43 64 75 10.3109/08916930903374634 20001423 PMC3660107

[B41] Dustin M Cooper J The immunological synapse and the actin cytoskeleton: molecular hardware for T cell signaling Nat Immunol 2101 1 23 29 10881170 10.1038/76877

[B42] Zhanga J Rubioa V Zheng S Shi Z Knockdown of OLA1, a regulator of oxidative stress response, inhibits motility and invasion of breast cancer cells J Zhejiang Univ Sci B 2009 10 11 796 804 10.1631/jzus.B0910009 19882753 PMC2772883

[B43] van Loo PF Bouwman P Ling K Middendorp G Suske G Grosveld F Dzierzak E Philipsen S Hendriks RW Impaired hematopoiesis in mice lacking the transcription factor Sp3 Blood 2003 102 3 858 866 10.1182/blood-2002-06-1848 12676787

[B44] Nemes Z Steinert P Bricks and mortar of the epidermal barrier Exp Mol Med 1999 31 5 19 10.1038/emm.1999.2 10231017

[B45] Champliaud M Viel A Baden H The Expression of Vitamin D-Upregulated Protein 1 in Skin and its Interaction with Sciellin in Cultured Keratinocytes J Invest Dermatol 2003 121 4 781 785 10.1046/j.1523-1747.2003.12539.x 14632196

[B46] Jeon J Yoon S Choi I JW C Vitamin D3 upregulated protein 1 (VDUP1) is a regulator for redox signaling and stress-mediated diseases J Dermatol 2006 33 662 669 10.1111/j.1346-8138.2006.00156.x 17040494

[B47] Tao K Wu C Wu K Li W Han G Shuai X Wang G Quantitative analysis of promoter methylation of the EDNRB gene in gastric cancer Med Oncol 2012 29 107 112 10.1007/s12032-010-9805-8 21264540

[B48] Krzewski K Gil-Krzewska A Watts J Stern JN Strominger JL VAMP4-and VAMP7-expressing vesicles are both required for cytotoxic granule exocytosis in NK cells Eur J Immunol 2011 41 11 3323 3329 10.1002/eji.201141582 21805468 PMC3438144

[B49] Zhang C De Koning DJ Hernandez-Sanchez J Haley CS Williams JL Wiener P Mapping of multiple quantitative trait loci affecting bovine spongiformencephalopathy Genetics 2004 167 4 1863 1872 10.1534/genetics.104.026401 15342524 PMC1470995

[B50] Levi-Montalcini R Hamburger V Selective growth stimulating effects of mouse sarcoma on the sensory and sympathetic nervous system of the chick embryo J Exp Zool 1951 116 321 361 10.1002/jez.1401160206 14824426

[B51] Schulman NF Viitala SM de Koning DJ Virta J Maki-Tanila A Vilkki JH Quantitative trait loci for health traits in Finnish Ayrshire cattle J Dairy Sci 2004 87 2 443 449 10.3168/jds.S0022-0302(04)73183-5 14762087

[B52] Schnabel RD Sonstegard TS Taylor JF Ashwell MS Whole-genome scan to detect QTL for milk production, conformation, fertility andfunctional traits in two US Holstein families Anim Genet 2005 36 5 408 416 10.1111/j.1365-2052.2005.01337.x 16167984

