# Supplementary material for: Genome-wide association study of infectious bovine keratoconjunctivitis in Angus cattle
Source: BMC Genet. 2013 Mar 26;14:23. doi: 10.1186/1471-2156-14-23 (PMC3673868; doi:10.1186/1471-2156-14-23)
Supplement: Additional file 1 — Top thirty 1-Mb SNP windows from genome-wide association study of IBK classified in two, three or nine categories. [file 1471-2156-14-23-S1.pdf]

Table S1: Top thirty 1-Mb SNP windows from genome-wide association study of IBK classified in two categories. The SNP loci were based on *Bos taurus* genome assembly (UMD 3.1)

Chromosome	Mb	rs number of first SNP	Position of first SNP	rs number of last SNP	Position of last SNP	Proportion of genetic variance	Cumulative genetic variance	Number of SNP in window
13	14	rs29021773	14356314	rs109429649	14923596	1.42	1.42	11
23	47	rs42027357	47005648	rs42037482	47953939	1.05	2.46	20
12	53	rs108956311	53009331	rs43705367	53986983	0.9	3.36	23
2	22	rs41642303	22087692	rs110857971	22768217	0.89	4.25	14
12	0	rs42556257	147119	rs42985713	947661	0.87	5.12	12
21	18	rs41639540	18012172	rs43726993	18975984	0.6	5.72	16
15	81	rs42804166	81067236	rs109347911	81964881	0.59	6.31	16
16	50	rs29018089	50046534	rs110161051	50994871	0.48	6.79	26
24	30	rs42045898	30028775	rs109000632	30881740	0.4	7.19	22
21	19	rs41966737	19002483	rs41640647	19983053	0.4	7.59	27
5	8	rs42632865	8038841	rs43429822	8978484	0.38	7.97	23
5	9	rs41652705	9126222	rs42071421	9950705	0.36	8.33	18
15	45	rs109032036	45104876	rs109057840	45968445	0.35	8.68	17
11	16	rs29009663	16020458	rs42870544	16983543	0.35	9.03	19
12	80	rs41579524	80058952	rs41660053	80978461	0.32	9.35	17
1	136	rs109611923	136029972	rs110921286	136996471	0.31	9.66	22
2	19	rs109448194	19105629	rs42270183	19965497	0.3	9.96	19
17	28	rs110372328	28001895	rs109478335	28923132	0.29	10.25	18
7	59	rs29026156	59019641	rs110705339	59974127	0.29	10.54	21
21	21	rs41582924	21017358	rs110219073	21983474	0.27	10.81	25
8	26	rs42211412	26062021	rs42794733	26971008	0.26	11.08	23
1	52	rs41600017	52030982	rs110575443	52979497	0.26	11.34	20
1	110	rs41611758	110037865	rs110290496	110982956	0.25	11.59	16
27	20	rs109309630	20067064	rs109100509	20962310	0.25	11.84	13
3	100	rs109119926	100009142	rs42676399	100982336	0.25	12.09	22
4	67	rs109413450	67007235	rs109462011	67880668	0.24	12.33	15
2	78	rs42630301	78028191	rs109611585	78993931	0.24	12.57	27
12	62	rs43418798	62028176	rs42495895	62990561	0.24	12.81	24
28	45	rs110078504	45075270	rs42157158	45997628	0.23	13.05	23
2	114	rs41608876	114008133	rs42466843	114986911	0.23	13.28	27

Table S2: Top thirty 1-Mb SNP windows from genome-wide association study of IBK classified in three categories. The SNP loci were based on *Bos taurus* genome assembly (UMD 3.1)

Chromosome	Mb	rs number of first SNP	Position of first SNP	rs number of last SNP	Position of last SNP	Proportion of genetic variance	Cumulative genetic variance	Number of SNP in window
23	47	rs42027357	47005648	rs42037482	47953939	2.62	2.62	20
12	53	rs108956311	53009331	rs43705367	53986983	1.01	3.63	23
22	57	rs109997561	57035240	rs110947166	57800571	0.82	4.45	15
12	0	rs42556257	147119	rs42985713	947661	0.66	5.11	12
5	9	rs41652705	9126222	rs42071421	9950705	0.63	5.75	18
23	50	rs42034499	50009589	rs109754466	50971212	0.52	6.27	23
13	14	rs29021773	14356314	rs109429649	14923596	0.5	6.77	11
1	112	rs110853931	112039035	rs41573010	112994639	0.46	7.24	17
2	19	rs109448194	19105629	rs42270183	19965497	0.45	7.69	19
16	50	rs29018089	50046534	rs110161051	50994871	0.43	8.12	26
21	7	rs109663123	7070882	rs110755620	7931963	0.41	8.53	16
15	42	rs109302360	42032171	rs109587766	42960757	0.37	8.9	22
15	22	rs109117719	22026329	rs41749166	22990151	0.36	9.26	19
2	76	rs41616196	76035542	rs41586870	76987725	0.35	9.61	25
1	109	rs42283451	109007793	rs41619077	109869888	0.35	9.96	23
11	16	rs29009663	16020458	rs42870544	16983543	0.34	10.3	19
15	45	rs109032036	45104876	rs109057840	45968445	0.34	10.64	17
5	71	rs109971012	71045870	rs41648982	71978791	0.33	10.97	18
23	43	rs41589780	43047899	rs42033912	43984482	0.33	11.3	19
1	29	rs43225355	29004968	rs43228767	29917811	0.33	11.62	21
4	36	rs42923240	36015624	rs29020436	36966070	0.32	11.95	21
25	30	rs110072941	30008822	rs109855978	30927675	0.31	12.26	23
8	108	rs110664854	108055243	rs41661224	108976660	0.31	12.57	19
7	1	rs109288175	1009369	rs110769696	1983458	0.3	12.87	21
20	26	rs110284833	26302807	rs41601571	26942991	0.3	13.17	11
2	22	rs41642303	22087692	rs110857971	22768217	0.28	13.45	14
5	105	rs42655314	105045500	rs109280888	105994337	0.27	13.72	20
22	58	rs42282429	58010164	rs110437833	58998502	0.27	13.99	27
21	19	rs41966737	19002483	rs41640647	19983053	0.26	14.26	27
22	4	rs29020896	4046850	rs109273661	4994019	0.26	14.52	15

Table S3: Top thirty 1-Mb SNP windows from genome-wide association study of IBK classified in nine categories. The SNP loci were based on *Bos taurus* genome assembly (UMD 3.1)

Chromosome	Mb	rs number of first SNP	Position of first SNP	rs number of last SNP	Position of last SNP	Proportion of genetic variance	Cumulative genetic variance	Number of SNP in window
10	36	rs109494217	36024341	rs41584997	36978160	1.23	1.23	19
12	53	rs108956311	53009331	rs43705367	53986983	1.11	2.34	23
1	32	rs43712972	32036293	rs43712998	32961952	0.96	3.31	19
22	57	rs109997561	57035240	rs110947166	57800571	0.92	4.23	15
23	47	rs42027357	47005648	rs42037482	47953939	0.65	4.88	20
17	28	rs110372328	28001895	rs109478335	28923132	0.64	5.51	18
1	31	rs110136403	31013633	rs43225084	31991587	0.51	6.02	16
11	68	rs41569387	68085486	rs41609923	68972281	0.47	6.49	15
5	71	rs109971012	71045870	rs41648982	71978791	0.46	6.95	18
13	62	rs110005922	62069793	rs110767129	62955647	0.45	7.4	19
5	72	rs41590781	72023321	rs109435449	72982750	0.44	7.85	21
1	112	rs110853931	112039035	rs41573010	112994639	0.43	8.28	17
12	62	rs43418798	62028176	rs42495895	62990561	0.39	8.67	24
13	14	rs29021773	14356314	rs109429649	14923596	0.37	9.04	11
1	110	rs41611758	110037865	rs110290496	110982956	0.36	9.4	16
21	18	rs41639540	18012172	rs43726993	18975984	0.35	9.75	16
24	30	rs42045898	30028775	rs109000632	30881740	0.35	10.1	22
8	61	rs41656490	61014570	rs41656448	61977595	0.35	10.44	26
3	94	rs109702825	94017640	rs110245538	94980177	0.35	10.79	17
2	22	rs41642303	22087692	rs110857971	22768217	0.32	11.12	14
4	120	rs110610134	120082962	rs110007192	120641946	0.32	11.44	14
8	0	rs109060553	438699	rs41255439	943817	0.31	11.75	16
21	19	rs41966737	19002483	rs41640647	19983053	0.29	12.05	27
2	116	rs41643747	116064542	rs41613018	116998234	0.29	12.34	20
8	51	rs110637350	51024542	rs29022887	51948998	0.29	12.63	24
15	81	rs42804166	81067236	rs109347911	81964881	0.28	12.91	16
1	24	rs29011682	24001042	rs29017639	24982221	0.28	13.19	24
2	19	rs109448194	19105629	rs42270183	19965497	0.27	13.46	19
15	41	rs109788031	41169953	rs41617498	41971248	0.27	13.73	17
1	45	rs41602958	45067210	rs42582276	45957974	0.26	13.99	20
